# Age‐specific over‐the‐top anterior cruciate ligament (ACL) reconstruction and lateral extra‐articular tenodesis (LET) in skeletally immature patients: No major complications during early follow‐up

**DOI:** 10.1002/jeo2.70852

**Published:** 2026-07-28

**Authors:** Ashraf Hantouly, Claudio Rossi, Bruna Cascone, Gian Andrea Lucidi, Lika Dzidzishvili, Kyle Borque, Stefano Zaffagnini, Alberto Grassi

**Affiliations:** ^1^ McMaster University Hamilton Ontario Canada; ^2^ Clinica Ortopedica E Traumatologica II, IRCCS Istituto Ortopedico Rizzoli Bologna Italy; ^3^ Dipartimento di Scienze Biomediche e Neuromotorie (DIBINEM) Università di Bologna Bologna Italy; ^4^ Hospital Universitari Germans Trias i Pujol Universidad Autónoma de Barcelona Barcelona Spain; ^5^ Houston Methodist Hospital Houston Texas USA

**Keywords:** ACL, lateral extra‐articular tenodesis, over‐the‐top, physeal sparing, skeletal age, skeletally immature

## Abstract

**Purpose:**

To assess the rate of early complications and reoperation after anterior cruciate ligament reconstruction (ACLR) using an over‐the‐top technique with lateral extra‐articular tenodesis (LET) in skeletally immature patients.

**Methods:**

This prospective case series included all skeletally immature patients undergoing ACLR with an over‐the‐top technique and LET augmentation between June 2022 and August 2025, performed by a single surgeon. Tibial tunnel strategy (extra‐physeal, supra‐physeal or trans‐physeal) was selected based on remaining growth. Skeletal immaturity was defined by the presence of open physes on knee magnetic resonance imaging. All patients had a minimum follow‐up of 9 months. The primary outcome was the rate of complications and reoperations within the first 9 post‐operative months, selected to capture events occurring during the rehabilitation phase prior to return to sport. Secondary outcomes included patient characteristics, sports activity, surgical technique and associated meniscal injuries.

**Results:**

Eighty‐four patients (mean age 14.7 ± 1.7 years; 77% male) were included, with a mean follow‐up of 20.0 ± 9.7 months. Surgical techniques included extra‐physeal (12%), supra‐physeal (48%) and trans‐physeal (40%) approaches. Concomitant meniscal lesions were treated in 71 patients. No major complications were observed. Minor complications occurred in 20.2% of patients, with similar rates across techniques. The most common events were transient stiffness (9.5%), early swelling/fever (8.3%), delayed wound healing (7.4%) and hardware irritation (2.4%). Reoperations occurred in 4.7% of cases, all before return to sport, and included hardware removal, wound debridement and joint lavage. No deep infections or septic arthritis were reported.

**Conclusion:**

ACLR using an over‐the‐top technique with LET in skeletally immature patients was not associated with major complications during the early postoperative period. Minor complications were observed in approximately one‐fifth of patients and were generally self‐limiting or managed without long‐term consequences. Longer follow‐up is required to evaluate growth‐related outcomes and graft survivorship.

**Level of Evidence:**

Level IV, case series.

**Abbreviations**
ACL: anterior cruciate ligament
ACLR: anterior cruciate ligament reconstruction
CRP: C‐reactive protein
ESR: erythrocyte sedimentation rate
LCL: lateral collateral ligament
LET: lateral extra‐articular tenodesis
LMORT: lateral meniscus oblique root treat
MCL: medial collateral ligament
MRI: magnetic resonance imaging
MTPF: menisco‐tibio‐popliteus‐fibular complex
MUA: manipulation under anaesthesia
PCL: posterior cruciate ligament
ROM: range of motion
RTP: return to play

## INTRODUCTION

Anterior cruciate ligament (ACL) injuries are among the most common and significant knee injuries in young athletes, with substantial implications for knee stability and long‐term athletic participation. Over the past decades, the incidence of ACL injuries and related publications has increased markedly [1, 29, 34]. This rise in injury incidence is largely attributed to greater year‐round sports participation, early specialization and higher training demands at younger ages [29, 34]. It is estimated that the incidence of ACL tears in skeletally immature patients ranges from 0.11 to 2.42 per 10,000, with a clear age‐related increase between 8 and 14 years [13]. Furthermore, data from Scandinavian registries demonstrate annual incidences of 76 per 100,000 in girls and 46 per 100,000 in boys, highlighting both the growing prevalence and the sex‐specific vulnerability of this population [17].

Historically, conservative management was the preferred treatment strategy in skeletally immature patients due to concerns about physeal injury during surgery, which could result in growth arrest, leg‐length discrepancies or angular deformities. However, advances in understanding injury patterns and long‐term outcomes have shifted this paradigm [22]. Multiple studies have demonstrated that nonoperative treatment or delayed surgical intervention is associated with higher rates of secondary intra‐articular damage, particularly meniscal tears and cartilage lesions. Accordingly, the number of paediatric ACL reconstructions (ACLRs) has increased dramatically by more than 300% in recent years, reflecting a broader acceptance of early surgical intervention [7, 14, 30].

Despite this shift, the optimal management strategy for ACL injuries in skeletally immature patients remains an area of ongoing debate. Controversy persists not only regarding the choice between surgical and nonsurgical management but also in the details of the management: timing of surgery, graft choice and surgical technique, whether trans‐physeal, supra‐physeal or extra‐physeal. These decisions aim to balance two critical objectives: restoring knee stability to allow safe return to sport and minimizing the risk of iatrogenic physeal injury. Nonoperative management has consistently demonstrated inferior rates of return to preinjury athletic performance, further supporting the need for effective surgical strategies in this population [3].

A major concern in paediatric ACLR is the potential for complications. Growth disturbance remains one of the most feared outcomes, although its true incidence appears low when physeal‐respecting techniques are employed [10, 29, 31]. This has led to the development of a variety of surgical approaches designed to minimize physeal insult, including physeal‐sparing (extra‐physeal and supra‐physeal) techniques and carefully executed trans‐physeal reconstructions. In contrast, early post‐operative complications—such as stiffness, wound healing issues, inflammatory reactions or hardware‐related symptoms—occur during the rehabilitation phase and may directly impact recovery progression, delay return to sport and influence patient tolerance to surgery [5, 23, 33].

In this context, adjunctive procedures such as lateral extra‐articular tenodesis (LET) have gained interest for their potential to enhance rotational stability and reduce graft strain, especially in high‐risk young athletes [16, 21, 29].

Despite the increasing use of combined intra‐ and extra‐articular techniques in skeletally immature patients, limited evidence specifically addresses early post‐operative safety and complication profiles of these procedures when applied through age‐specific surgical algorithms in skeletally immature patients. Most available studies primarily focus on mid‐ to long‐term outcomes, leaving the early post‐operative phase insufficiently characterized in this population [1, 29].

Therefore, given the increasing demand for paediatric ACLR and the importance of minimizing complications, this study was designed to evaluate the early safety and reoperation rates associated with ACLR using a single‐bundle over‐the‐top technique combined with LET in skeletally immature patients [18, 36, 38]. The hypothesis was that this technique would not be associated with a high rate of major complications or reoperations across different stages of skeletal immaturity.

## METHODS

### Study design

This study was reported with strict adherence to the Preferred Reporting of Case Series in Surgery (PROCESS) checklist for case series studies [26]. The study was approved by the Institutional Review Board (IRB) (Approval No. 380/2019/Oss/IOR) on May 22, 2019, and was conducted in accordance with the Declaration of Helsinki. Informed consent was obtained from all patients and/or their legal guardians.

All skeletally immature patients with either an open femoral or tibial physis on knee magnetic resonance imaging (MRI) who underwent ACLR using over‐the‐top technique with LET augmentation performed by a single surgeon (A. G.) between June 2022 and August 2025 were screened for eligibility. Patients with previous ipsilateral knee surgery, pre‐existing physeal bar, growth disturbance or previous fracture crossing the physis, concomitant major ligament injury (medial collateral ligament, lateral collateral ligament and posterior cruciate ligament), and those with incomplete clinical data were excluded. There was no restriction on concomitant meniscal pathologies.

Complications were categorized as major or minor based on clinical relevance, impact on patient recovery and need for surgical intervention, in accordance with previously described concepts in surgical literature, where complication severity is defined by clinical impact rather than the mere occurrence of a reoperation (e.g., Clavien–Dindo classification) [5, 6]. Accordingly, low‐morbidity procedures not affecting graft integrity or long‐term outcomes were classified as minor complications.

Major complications were defined as events associated with significant morbidity or structural failure, including deep infection, graft failure, growth disturbance, neurovascular injury or arthrofibrosis requiring manipulation under anaesthesia (MUA).

Minor complications were defined as self‐limiting or conservatively managed conditions—including postoperative swelling or fever, delayed wound healing, transient stiffness and hardware irritation—or events requiring minor surgical procedures without structural consequences. These included hardware removal, arthroscopic lavage or wound debridement when not associated with graft failure, infection, growth disturbance or long‐term functional impairment, and when they did not result in a significant delay in rehabilitation or return to sport.

Indication for ACLR in this series of skeletally immature patients was based on the evaluation of several parameters that were critically assessed by the treating surgeon, such as skeletal age, presence of meniscal tears in the pre‐operative MRI and high‐grade (3+) rotatory laxity at pivot‐shift assessment. In the case when patients were initially treated conservatively elsewhere, the presence of recurrent instability episodes, pain or inability to return to sport participation were considered as well as indications for surgery. Only patients with at least 9 months of follow‐up and who were fully disclosed to return to physical activity were included in the study.

### Surgical technique

All patients underwent ACLR with an over‐the‐top plus lateral tenodesis technique using hamstring tendons according to an algorithm based on skeletal age and remaining growth.

**Figure 1:**
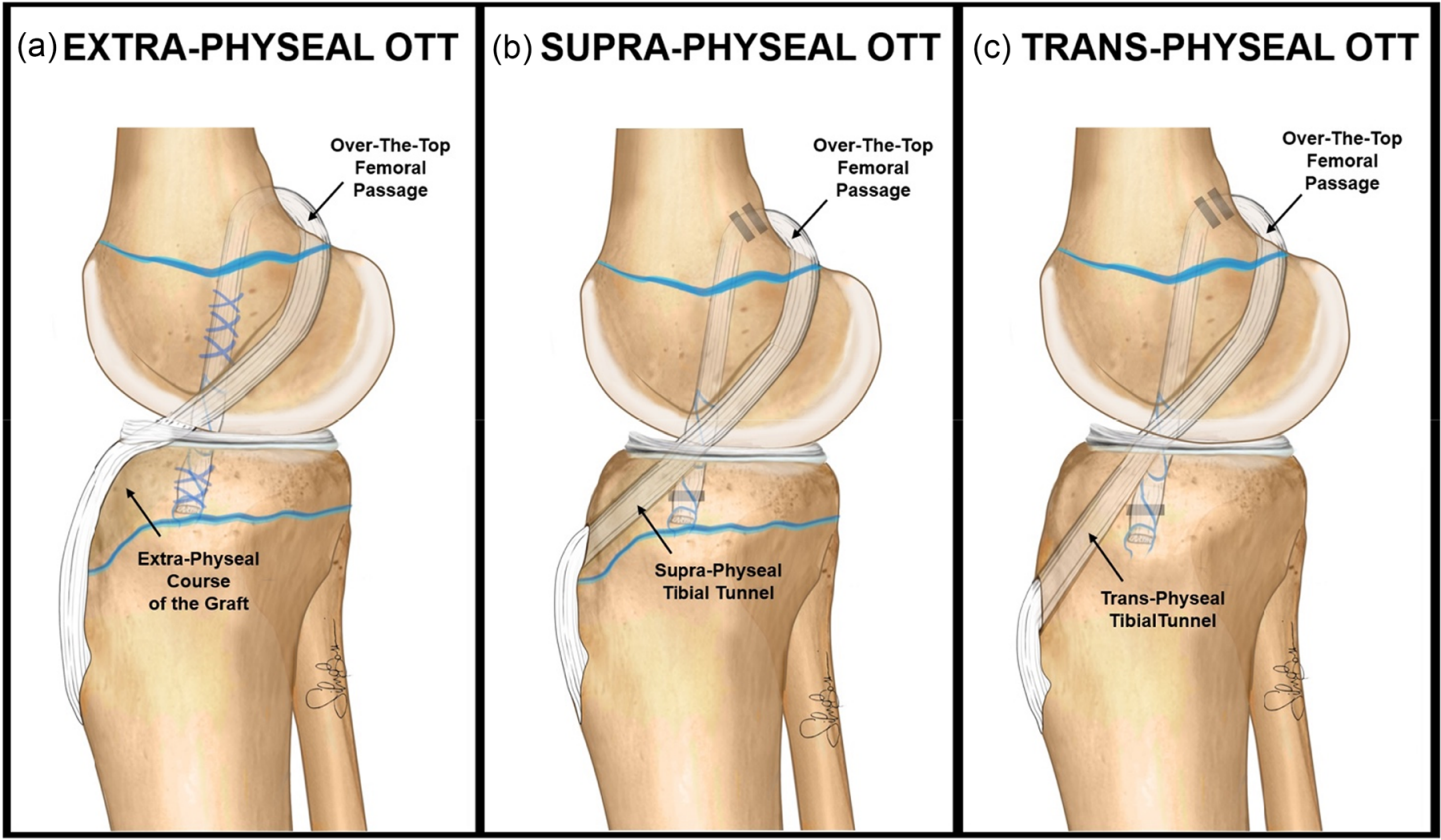
The 3 variants of the over‐the‐top technique according to skeletal age: ‘extra‐physeal’ passage of graft (a), ‘supra‐physeal’ tibial tunnel (b) and ‘trans‐physeal’ tibial tunnel (c).

In ‘prepubescent’ patients (males <12 years and females <10 years of skeletal age), an ‘extra‐physeal’ over‐the‐top technique was used. The hamstring graft was passed under the intermeniscal ligament before being secured first to the lateral femur and then to the tibia at Gerdy's tubercle utilizing periosteal sutures. No tunnels or hardware were used (Figure 1a) [20].

In ‘young adolescents’ (males 12–15 years and females 10–13 years of skeletal age), a ‘supra‐physeal’ over‐the‐top technique was used. In this case, a tibial tunnel was created proximally to the tibial physis (within the proximal tibial epiphysis) under fluoroscopic guidance, and the graft was secured with staples above both the femoral physis and the lateral tenodesis above the tibial physes (Figure 1b) [11].

In ‘older adolescents’ (males 16–18 years and females 14–16 years of skeletal age), a ‘trans‐physeal’ over‐the‐top technique was used, with the original technique as described in adults, with the tibial tunnel drilled through the closed or closing tibial physis (Figure 1c) [19].

Medial and lateral menisci were carefully inspected, palpated and mobilized with the probe in order to identify and assess possible lesions. Meniscal repair was performed with Truespan (DePuy) or Ultra Fast‐Fix (Smith and Nephew) all‐inside devices.

In the case of meniscal repair, an extension brace was prescribed for 1 month. No weight bearing for 1 month was applied in the case of root and lateral meniscus oblique root tears (LMORT), while partial weight bearing was allowed in the case of bucket handle, longitudinal and menisco‐tibio‐popliteus‐fibular (MTPF) complex tears. Passive range of motion exercises were initiated 2 days after surgery, restricting flexion to 90° for the first 2 weeks, and flexion until 120° was progressively allowed in the next 2 weeks. A stationary bike was allowed after 6 weeks and running and squatting after 3–4 months. Patients were advised to return to play (RTP) at least 9 months after surgery, depending on the type of sport.

### Patients' assessment

The pre‐operative and operative notes and post‐operative assessment records were reviewed. The variables of interest included age, sex, sports activity, concomitant knee pathologies, surgical technique, follow‐up, remaining growth, complications and reoperation. Two authors performed data collection, and any discrepancy was resolved with a discussion with the senior author.

All patients were followed by the treating surgeon after 2 weeks, 6 weeks, 3 months, 6 months and 9 months to monitor the post‐operative recovery and rehabilitation. Extension deficit was considered when the patient was not able to fully extend the operated knee. Flexion deficit was considered when the patient was not able to reach 90° of flexion at the 6‐week assessment. In the case of post‐operative swelling and fever, blood exams with white cell count, C‐reactive protein and erythrocyte sedimentation rate were performed and repeated after 3–5 days. Joint fluid collection and examination were performed in the case of suspected septic arthritis.

Hardware irritation was considered when the patient presented with a localized tenderness at the level of the distal femur or proximal tibia over the implant. In the case of swelling over the implants and persistent tenderness after 6 months, hardware removal was considered.

## RESULTS

### Patients' characteristics

A total of 101 skeletally immature patients underwent ACLR in the considered period. A total of 84 patients (83%) had at least 9 months of follow‐up and were disclosed for return to sport and thus were included in the present study.

The 84 patients included were mostly male (77%) and had an average age at surgery of 14.7 ± 1.7 years. Skeletal age was 14.3 ± 1.9 years, with 1.5 ± 1.8 years of remaining growth and 2 or more years in 32 cases (38%) (Table 1).

**Table 1:** Characteristics of the included patients.

Patients characteristics	
Demographic details	
Sex (M/F)	65 (77%)/19 (23%)
Age (years)	14.7 ± 1.7
Remaining knee growth	
<2 years	52 (62%)
≥2 years	32 (38%)
Meniscal lesions	71 (84%)
Medial	37 (44%)
Laterale	54 (64%)
Sport activity	
Soccer	53 (63%)
Basketball	12 (14%)
Volleyball	5 (6%)
Martial arts	3 4%)
Rugby	3 (4%)
Skii	3 (4%)
Motocross	1 (1%)
Dance	1 (1%)
Swimming	1 (1%)

Considering the surgical algorithm, 10 patients (12%) were treated with the ‘extra‐physeal’ technique, 40 patients (48%) with the ‘supra‐physeal’ technique and 34 patients (40%) with the ‘trans‐physeal’ technique. A total of 71 patients (84%) had a concomitant meniscal lesion that was treated with repair: 37 patients (44%) for a medial meniscus lesion and 54 patients (64%) for a lateral meniscus lesion. The average duration of the surgical procedure (skin to skin) was 63 ± 16 minutes (range: 39–104 min), with the longest surgical time for the ‘extra‐physeal’ (*p* = 0.0430).

### Complications and reoperations

At a minimum follow‐up of 9 months and at an average follow‐up of 20.0 ± 9.7 months, no major complications (0%) were reported. Seventeen patients (20.2%) presented a total of 21 minor complications: seven (8.3%) post‐operative swelling and fever within the first 3 weeks, four (7.4%) delayed wound healing of the hamstring harvesting incision, eight (9.5%) transient stiffness which were solved in all cases with intensive physiotherapy without surgery by the fourth post‐operative month, and two (2.4%) hardware irritation which required staple removal (Table 2).

**Table 2:** Patient characteristics and post‐operative outcomes according to the specific over‐the‐top technique.

Complications and reoperations based on over‐the‐top techniques				
	Extra‐physeal over‐the‐top	Supra‐physeal over‐the‐top	Trans‐physeal over‐the‐top	*p* Value
Number of patients	10 (12%)	40 (48%)	34 (40%)	
Age (years)	11.9 ± 0.9	14.2 ± 1.4	15.7 ± 0.9	<0.05*
Sex (M/F)	10 (100%)/0 (0%)	33 (83%)/7 (17%)	22 (65%)/12 (35%)	<0.05*
Mean remaining growth (years)	5.3 ± 0.5	1.6 ± 0.8	0.0 ± 0.0	<0.05*
Surgical time (min)	77 ± 11	63 ± 13	61 ± 18	<0.05*
Patients with minor complications	2 (20%)	7 (17%)	8 (23%)	n.s.
Stiffness	2 (20%)	3 (7%)	3 (9%)	n.s.
Delayed wound healing	1 (10%)	2 (5%)	1 (3%)	n.s.
Swelling and fever	0 (0%)	4 (10%)	3 (9%)	n.s.
Hardware irritation	0 (0%)	0 (0%)	2 (6%)	n.s.
Patients with reoperations	0 (0%)	2 (5%)	2 (6%)	n.s.
Hardware removal	0 (0%)	0 (0%)	2 (6%)	n.s.
Arthroscopic debridement	0 (0%)	1 (2%)	0 (0%)	n.s.
Wound debridement	0 (0%)	1 (2%)	0 (0%)	n.s.

Abbreviation: n.s., not significant.

* Significant values.

A total of four patients (4.7%) underwent a re‐operation within the first‐post operative 9 months and before disclosure to return to sport: two staple removals (one on femoral side, one on tibial side), one wound debridement and closure for a delayed healing, and one joint lavage for post‐operative swelling. In both the latter cases, intra‐operative cultures were taken without any signs of infection. The other two patients underwent a surgical procedure after full RTP, which were not considered complications: one ACL revision for a traumatic graft re‐rupture 18 months after surgery (1%) and one partial medial meniscectomy after meniscal repair 22 months after surgery (1%).

## DISCUSSION

The main finding of this study is that ACLR in skeletally immature patients using an over‐the‐top technique with LET augmentation was not associated with major complications during the evaluated period. Across all three techniques—extra‐physeal, supra‐physeal and trans‐physeal—no major complications were observed, and no statistically significant differences were identified in the incidence of minor complications or reoperations. Overall, minor complications occurred in 20.2% of patients, and the reoperation rate was 4.7%. Most minor complications were transient or managed without long‐term consequences, and no deep infections, graft failures, growth disturbances or arthrofibrosis requiring MUA were observed during the study period.

These findings are in line with the previously published data. Several studies have reported low rates of complications and growth disturbances following ACLR in skeletally immature patients [4, 9, 12, 15, 24]. While the rate of post‐operative stiffness in this study appears higher when examined in isolation (9.5%), it is important to contextualize these findings. Cruz et al. reported an arthrofibrosis rate of 1.9%, but only cases requiring MUA were included in their definition [5]. In contrast, although some patients in the present series showed signs of stiffness, none required MUA and all improved with dedicated physiotherapy within 4 months from surgery. When differences in definitions and reporting standards are considered, the results of this study remain consistent with current evidence and highlight the importance of standardized reporting of complications in this population.

Infections following paediatric ACLR are rare, and this is supported by this study [32]. No true postoperative infections were found, although two patients underwent reoperation for persistent swelling and delayed wound healing. Neither represented deep or superficial infections confirmed by cultures. These observations align with Knorr et al., who reported one infection among 74 patients [23], and Wall et al., who described postoperative skin reactions rather than true infections, none of which required operative intervention [33]. The swelling rate of 8.3% in this study likely reflects the exaggerated inflammatory response typical in younger patients; importantly, these episodes resolved with observation and conservative management. Together, these data reinforce that infectious complications after paediatric ACLR remain uncommon. The absence of major complications and infections could also be related to the short surgical timing [28]. In fact, despite a physeal sparing approach being used, the average surgical time was nearly one hour, with just less than 15 min increase in prepubescent patients.

Although the follow‐up of this study is short to detect angular deformities and leg length discrepancy, long‐term literature consistently shows that complications and growth disturbances are infrequent following both physeal‐sparing ACLR when performed with careful respect for physeal anatomy [4, 9, 10, 12, 15, 24, 29, 34]. Pierce et al.'s systematic review of 942 patients undergoing ACLR in skeletally immature patients with more than 4 years of follow‐up demonstrated no significant difference in growth disturbance or graft survivorship between the different techniques [28]. Another systematic review of 935 patients with a mean age of 13 years and a follow‐up of 40 months reported a leg‐length discrepancy rate of only 1.8% [12]. While longer‐term evaluation of this series is still needed, early results are reassuring and consistent with established evidence [27].

A major finding in this study was the high prevalence of concomitant meniscal injuries, with 84% of patients presenting with either medial or lateral meniscal pathology. Lateral meniscal tears were more frequent (54%) than medial tears (44%), a pattern well‐documented in paediatric ACL injuries. Dumont et al. reported lateral meniscal lesions in more than half of their paediatric cohort regardless of injury chronicity [8]. Similarly, a cohort study by Matava on 748 patients demonstrated that 423 patients (57%) had 283 lateral meniscal tears, 69 medial meniscal tears and 71 both a lateral and a meniscal tear [25]. The absence of major complications despite the high prevalence of concomitant meniscal tears supports the feasibility of a comprehensive joint‐preserving approach in skeletally immature patients with appropriate surgical techniques [35].

The role of physeal‐sparing techniques in the older adolescent subgroup warrants discussion, as these patients were approaching skeletal maturity and frequently demonstrated a closed or closing tibial physis. The mean age of patients in this subgroup was 15.7 years, suggesting that many could have been considered candidates for a standard ACLR technique. However, these patients were included in the present algorithm because a residual open femoral physis was still visible on MRI, and they therefore met the study definition of skeletal immaturity. The rationale of the proposed algorithm was to encompass the entire spectrum of skeletally immature patients, ranging from prepubescent children to adolescents approaching skeletal maturity while still demonstrating MRI evidence of open physes. Since the over‐the‐top approach has been routinely adopted across adolescent, adult and professional athletic populations at the authors' institution, a standard transtibial or anteromedial portal ACLR was not incorporated into the proposed algorithm [2, 5, 8, 19, 23, 25, 28, 33, 36, 37]. Furthermore, applying a femoral physeal‐sparing technique in these patients may theoretically further minimize physeal violation in patients with residual growth potential.

This study offers some methodological strengths. All procedures were performed by a single experienced surgeon using a consistent surgical technique, reducing variability in operative decision‐making and execution. The prospective design with no patients lost to follow‐up strengthens the reliability of the dataset. Additionally, the series was homogeneous, consisting exclusively of skeletally immature patients receiving the same graft type and the same surgical technique, which minimizes confounding related to graft selection and surgical technique.

However, limitations must also be acknowledged. Although the sample size is modest, it remains one of the larger series evaluating these techniques within this specific population. The follow‐up duration is short, limiting the ability to detect mid‐term to late complications such as graft failure, angular deformities and leg‐length discrepancies. Additionally, there was no control group. Finally, the absence of functional outcome scores also restricts the ability to correlate complication profiles with patient‐reported recovery. However, it was the aim of the study to investigate the early safety and post‐operative patient tolerance.

## CONCLUSIONS

ACLR using an age‐specific over‐the‐top approach with LET augmentation in skeletally immature patients was not associated with major complications during early follow‐up. Minor complications occurred in 20.2% of patients and reoperations in 4.7% of cases, with most events being transient or managed without long‐term consequences or delay in return to sport. No deep infections, graft failures, growth disturbances or arthrofibrosis requiring MUA were observed during the study period. Longer follow‐up is required to evaluate graft survivorship, growth‐related outcomes and late complications.

## AUTHOR CONTRIBUTIONS

Alberto Grassi conceived and designed the study, performed the surgical procedures, supervised the study, interpreted the data and drafted the manuscript. Claudio Rossi and Bruna Cascone collected and curated the data. Gian Andrea Lucidi performed the statistical analysis and contributed to data interpretation. Ashraf Hantouly, Claudio Rossi, Lika Dzidzishvili, Kyle Borque, Stefano Zaffagnini and Alberto Grassi critically interpreted the findings and revised the manuscript for important intellectual content. All authors approved the final version of the manuscript.

## CONFLICT OF INTEREST STATEMENT

Alberto Grassi declares the following conflict of interest: Paid courses and faculty member for DePuy and Smith and Nephew. The remaining authors declare no conflict of interest.

## FUNDING INFORMATION

The authors have no funding to report.

## ETHICS STATEMENT

This study was approved by the Institutional Review Board (IRB) under protocol number CE‐AVEC 380/2019/Oss/IOR, protocol number 0006881. All patients' legal guardians provided written informed consent for the use of their data in scientific research, in accordance with the Declaration of Helsinki.

## Data Availability

The data that support the findings of this study are available from the corresponding author upon reasonable request.

## References

[1] Alzobi OZ , Almannai H , Hantouly A , Salman LA , Ahmed AF , Alkhelaifi KA , et al. Anterior cruciate ligament reconstruction with lateral extra‐articular augmentation: a bibliometric analysis of the top 100 cited articles. J Knee Surg. 2025;38(11):580–591. 40368406 10.1055/a-2608-0220

[2] Bonanzinga T , Grassi A , Altomare D , Lucidi GA , Macchiarola L , Zaffagnini S , et al. High return to sport rate and few re‐ruptures at long term in professional footballers after anterior cruciate ligament reconstruction with hamstrings. Knee Surg Sports Traumatol Arthrosc. 2022;30(11):3681–3688. 35451640 10.1007/s00167-022-06944-1

[3] Cancino B , Muñoz C , Tuca MJ , Birrer EAM , Sepúlveda MF . Anterior cruciate ligament rupture in skeletally immature patients. J Am Acad Orthop Surg Glob Res Rev. 2022;6(5):e21.00166. 10.5435/JAAOSGlobal-D-21-00166 PMC10531303 35588096

[4] Courvoisier A , Grimaldi M , Plaweski S . Good surgical outcome of transphyseal ACL reconstruction in skeletally immature patients using four‐strand hamstring graft. Knee Surg Sports Traumatol Arthrosc. 2011;19(4):588–591. 20890694 10.1007/s00167-010-1282-2

[5] Cruz AI , Fabricant PD , McGraw M , Rozell JC , Ganley TJ , Wells L . All‐epiphyseal ACL reconstruction in children: review of safety and early complications. J Pediatr Orthop. 2017;37(3):204–209. 26192883 10.1097/BPO.0000000000000606

[6] Dindo D , Demartines N , Clavien P‐A . Classification of surgical complications: a new proposal with evaluation in a cohort of 6336 patients and results of a survey. Ann Surg. 2004;240(2):205–213. 15273542 10.1097/01.sla.0000133083.54934.ae PMC1360123

[7] Dodwell ER , Lamont LE , Green DW , Pan TJ , Marx RG , Lyman S . 20 Years of pediatric anterior cruciate ligament reconstruction in New York state. Am J Sports Med. 2014;42(3):675–680. 24477820 10.1177/0363546513518412

[8] Dumont GD , Hogue GD , Padalecki JR , Okoro N , Wilson PL . Meniscal and chondral injuries associated with pediatric anterior cruciate ligament tears: relationship of treatment time and patient‐specific factors. Am J Sports Med. 2012;40(9):2128–2133. 22729621 10.1177/0363546512449994

[9] Ebert JR , Sobhi S , Annear PT . Transphyseal ACL reconstruction and tenodesis in skeletally immature patients demonstrates encouraging clinical scores, without growth disturbance, excessive laxity or re‐injury. J Orthop. 2024;52:55–60. 38435316 10.1016/j.jor.2024.02.028 PMC10901687

[10] Elnewishy A , Elgamal M , Shah S , Hamada A , Ali MA , Noureldin M , et al. Outcomes of all‐epiphyseal anterior cruciate ligament (ACL) reconstruction in skeletally immature patients: a systematic review and meta‐analysis. Cureus. 2025;17(10):e94359. 41220455 10.7759/cureus.94359 PMC12600025

[11] Fernández‐Comparini T , Irarrázaval S , Fernández Schlein F , Besa P , Lira MJ , Vidal C , et al. Optimizing tunnel placement for lateral extra‐articular procedures in pediatric epiphyseal anterior cruciate ligament reconstruction: a three‐dimensional simulation study of physeal damage and safe zone identification. J ISAKOS. 2026;19:101129. 42106007 10.1016/j.jisako.2026.101129

[12] Frosch K‐H , Stengel D , Brodhun T , Stietencron I , Holsten D , Jung C , et al. Outcomes and risks of operative treatment of rupture of the anterior cruciate ligament in children and adolescents. Arthroscopy. 2010;26(11):1539–1550. 21035009 10.1016/j.arthro.2010.04.077

[13] Funahashi KM , Moksnes H , Maletis GB , Csintalan RP , Inacio MCS , Funahashi TT . Anterior cruciate ligament injuries in adolescents with open physis: effect of recurrent injury and surgical delay on meniscal and cartilage injuries. Am J Sports Med. 2014;42(5):1068–1073. 24634449 10.1177/0363546514525584

[14] Fury MS , Paschos NK , Fabricant PD , Anderson CN , Busch MT , Chambers HG , et al. Assessment of skeletal maturity and postoperative growth disturbance after anterior cruciate ligament reconstruction in skeletally immature patients: a systematic review. Am J Sports Med. 2022;50(5):1430–1441. 33984243 10.1177/03635465211008656

[15] Gaulrapp HM , Haus J . Intraarticular stabilization after anterior cruciate ligament tear in children and adolescents: results 6 years after surgery. Knee Surg Sports Traumatol Arthrosc. 2006;14(5):417–424. 16402220 10.1007/s00167-005-0698-6

[16] Getgood AMJ , Bryant DM , Litchfield R , Heard M , McCormack RG , Rezansoff A , et al. Lateral extra‐articular tenodesis reduces failure of hamstring tendon autograft anterior cruciate ligament reconstruction: 2‐year outcomes from the STABILITY study randomized clinical trial. Am J Sports Med. 2020;48(2):285–297. 31940222 10.1177/0363546519896333

[17] Granan L‐P , Forssblad M , Lind M , Engebretsen L . The Scandinavian ACL Registries 2004–2007: baseline epidemiology. Acta Orthop. 2009;80(5):563–567. 19916690 10.3109/17453670903350107 PMC2823321

[18] Grassi A , Borque KA , Laughlin MS , Tao MA , Zaffagnini S . Age‐specific over‐the‐top techniques for physeal sparing anterior cruciate ligament (ACL) reconstruction in skeletally immature patients: current concepts for prepubescents to older adolescents. Knee Surg Sports Traumatol Arthrosc. 2025;33(10):3510–3522. 39901868 10.1002/ksa.12607 PMC12459316

[19] Grassi A , Pizza N , Macchiarola L , Lucidi GA , Stefanelli F , Dal Fabbro G , et al. Over‐the‐top anterior cruciate ligament (ACL) reconstruction plus lateral plasty with hamstrings in high‐school athletes: results at 10 years. Knee. 2021;33:226–233. 34717094 10.1016/j.knee.2021.10.004

[20] Grassi A , Zaffagnini S , Borque K . Extraphyseal anterior cruciate ligament reconstruction and lateral tenodesis using hamstrings for prepubescent patients: the WHAT technique (without hardware and tunnels). Arthrosc Tech. 2025;14(8):103708. 40936581 10.1016/j.eats.2025.103708 PMC12420572

[21] Hantouly AT , Ahmed AF , Fermin TM , Macchiarola L , Sideris V , Papakostas E , et al. Short‐term outcomes of anterior cruciate ligament reconstruction with or without lateral tenodesis or anterolateral ligament reconstruction: a retrospective cohort. Int Orthop. 2023;47(12):2991–2999. 37632528 10.1007/s00264-023-05931-6 PMC10673961

[22] Heyworth BE , Liotta ES , Kay J , Williams KA , Kocher MS , Micheli LJ , et al. Physeal sparing combined extra‐articular/intra‐articular iliotibial band ACL reconstruction in children: a long‐term strength, dynamic balance, and functional analysis. Orthop J Sports Med. 2026;14(4):23259671261430745. 42046833 10.1177/23259671261430745 PMC13110322

[23] Knörr J , Sales de Gauzy J , Doménech P , Sánchez M , Soldado F , Barrios C . Anterior cruciate ligament reconstruction in skeletally immature patients using an all‐epiphyseal technique: a prospective cohort study. Orthop J Sports Med. 2025;13(3):23259671251322771. 40160290 10.1177/23259671251322771 PMC11954573

[24] Kocher MS , Smith JT , Zoric BJ , Lee B , Micheli LJ . Transphyseal anterior cruciate ligament reconstruction in skeletally immature pubescent adolescents. J Bone Joint Surg Am. 2007;89(12):2632–2639. 18056495 10.2106/JBJS.F.01560

[25] Matava MJ , Gibian JT , Hutchinson LE , Miller PE , Milewski MD , Pennock AT , et al. Factors associated with meniscal and articular cartilage injury in the PLUTO cohort. Am J Sports Med. 2023;51(6):1497–1505. 37014299 10.1177/03635465231164952

[26] Mathew G , Sohrabi C , Franchi T , Nicola M , Kerwan A , Agha R , et al. Preferred Reporting Of Case Series in Surgery (PROCESS) 2023 Guidelines. Int J Surg. 2023;109(12):3760–3769. 37988417 10.1097/JS9.0000000000000940 PMC10720832

[27] Ntagiopoulos P , Kalinterakis G , Pozzi P , Fligkos D , Themistocleous G , Themistokleous S , et al. All‐epiphyseal, all‐inside ACL reconstruction yields high return‐to‐sport and minimal growth‐related complications at mid‐ to long‐term follow‐up in skeletally immature patients. J Exp Orthop. 2026;13(1):e70671. 41768533 10.1002/jeo2.70671 PMC12936850

[28] Pierce TP , Issa K , Festa A , Scillia AJ , McInerney VK . Pediatric anterior cruciate ligament reconstruction: a systematic review of transphyseal versus physeal‐sparing techniques. Am J Sports Med. 2017;45(2):488–494. 27045088 10.1177/0363546516638079

[29] Roberti di Sarsina T , Macchiarola L , Signorelli C , Grassi A , Raggi F , Marcheggiani Muccioli GM , et al. Anterior cruciate ligament reconstruction with an all‐epiphyseal ‘over‐the‐top’ technique is safe and shows low rate of failure in skeletally immature athletes. Knee Surg Sports Traumatol Arthrosc. 2019;27(2):498–506. 30209520 10.1007/s00167-018-5132-y

[30] Sasaki S , Sasaki E , Kimura Y , Yamamoto Y , Tsuda E , Ishibashi Y . Clinical outcomes and postoperative complications after all‐epiphyseal double‐bundle ACL reconstruction for skeletally immature patients. Orthop J Sports Med. 2021;9(11):23259671211051308. 34778480 10.1177/23259671211051308 PMC8586179

[31] Shanmugaraj A , de Sa D , Skelly MM , Duong A , Simunovic N , Musahl V , et al. Primary allograft ACL reconstruction in skeletally immature patients—a systematic review of surgical techniques, outcomes, and complications. J Knee Surg. 2019;32(7):673–685. 29991077 10.1055/s-0038-1666833

[32] Sinha R , Pelt RV , Green DW , Shea KG , Niu EL , Saper MG , et al. Increased operative time is an independent risk factor for surgical complications following isolated anterior cruciate ligament reconstruction in skeletally immature patients. J Exp Orthop. 2026;13:e70759. 42164840 10.1002/jeo2.70759 PMC13185210

[33] Wall EJ , Ghattas PJ , Eismann EA , Myer GD , Carr P . Outcomes and complications after all‐epiphyseal anterior cruciate ligament reconstruction in skeletally immature patients. Orthop J Sports Med. 2017;5(3):2325967117693604. 28451597 10.1177/2325967117693604 PMC5400138

[34] Wong SE , Feeley BT , Pandya NK . Comparing outcomes between the over‐the‐top and all‐epiphyseal techniques for physeal‐sparing ACL reconstruction: a narrative review. Orthop J Sports Med. 2019;7(3):2325967119833689. 30944841 10.1177/2325967119833689 PMC6440065

[35] Yen Y‐M , Fabricant PD , Richmond CG , Dingel AB , Milewski MD , Ellis HB , et al. Proximity of the neurovascular structures during all‐inside lateral meniscal repair in children: a cadaveric study. J Exp Orthop. 2018;5(1):50. 30564981 10.1186/s40634-018-0166-0 PMC6298911

[36] Zaffagnini S , Lucidi GA , Macchiarola L , Agostinone P , Neri MP , Marcacci M , et al. The 25‐year experience of over‐the‐top ACL reconstruction plus extra‐articular lateral tenodesis with hamstring tendon grafts: the story so far. J Exp Orthop. 2023;10(1):36. 37005946 10.1186/s40634-023-00599-8 PMC10067780

[37] Zaffagnini S , Marcheggiani Muccioli GM , Grassi A , Roberti di Sarsina T , Raggi F , Signorelli C , et al. Over‐the‐top ACL reconstruction plus extra‐articular lateral tenodesis with hamstring tendon grafts: prospective evaluation with 20‐year minimum follow‐up. Am J Sports Med. 2017;45(14):3233–3242. 28922015 10.1177/0363546517723013

[38] Zaffagnini S , Mok YR , Grassi A , Muccioli GMM . Over‐the‐top plus lateral extra‐articular tenodesis anterior cruciate ligament reconstruction: ten tips for success. J Exp Orthop. 2026;13(2):e70785. 42255389 10.1002/jeo2.70785 PMC13238623

